# Ultrafast Optical Microscopy of Single Monolayer Molybdenum Disulfide Flakes

**DOI:** 10.1038/srep21601

**Published:** 2016-02-15

**Authors:** Minah Seo, Hisato Yamaguchi, Aditya D. Mohite, Stephane Boubanga-Tombet, Jean-Christophe Blancon, Sina Najmaei, Pulickel M. Ajayan, Jun Lou, Antoinette J. Taylor, Rohit P. Prasankumar

**Affiliations:** 1 Center for Integrated Nanotechnologies, Los Alamos National Laboratory, Los Alamos, NM, 87545, USA; 2 Sensor System Research Center, Korea Institute of Science and Technology, Seoul, Republic of Korea; 3 Materials Synthesis and Integrated Devices, Materials Physics and Applications Division, Los Alamos National Laboratory, Los Alamos, NM 87545, USA; 4 Physical Chemistry and Applied Spectroscopy, Chemistry Division, Los Alamos National Laboratory, Los Alamos, NM 87545, USA; 5 Department of Materials Science and NanoEngineering, Rice University, Houston, Texas 77005, USA; 6 Research Institute of Electrical Communication, Tohoku University, 2-1-1 Katahira, Aoba-Ku, Sendai 980-8577, Japan

## Abstract

We have performed ultrafast optical microscopy on single flakes of atomically thin CVD-grown molybdenum disulfide, using non-degenerate femtosecond pump-probe spectroscopy to excite and probe carriers above and below the indirect and direct band gaps. These measurements reveal the influence of layer thickness on carrier dynamics when probing near the band gap. Furthermore, fluence-dependent measurements indicate that carrier relaxation is primarily influenced by surface-related defect and trap states after above-bandgap photoexcitation. The ability to probe femtosecond carrier dynamics in individual flakes can thus give much insight into light-matter interactions in these two-dimensional nanosystems.

There has been an explosion of recent interest in the quasi-two-dimensional (2D) transition metal dichalcogenides (TMDCs), which are layered materials with strong in-plane covalent bonding, leading to atomically thin layers within their structure^1^. Their unique 2D geometry allows for the design and fabrication of complicated structures at desirable positions within optoelectronic devices, much more efficiently than other low-dimensional nanomaterials^1^,^2^. In particular, molybdenum disulfide (MoS_2_) has received much recent attention due to its excellent electronic^3^ and optical properties^4^,^5^. Most notably, the indirect bandgap in the bulk (~1.2 eV) changes to a direct bandgap (~1.9 eV) as the number of atomic layers is reduced to one, causing MoS_2_ monolayers to generate strong photoluminescence^4^,^5^,^6^,^7^. Therefore, unlike graphene, which does not have an intrinsic band gap^8^, TMDCs are especially promising for potential optoelectronic applications, such as photo-transistors^9^, photodetectors^10^, and electroluminescent devices^11^. Many of these applications will depend on a detailed knowledge of the optical properties and carrier relaxation dynamics, which can be elucidated by photoexciting carriers and probing their relaxation as a function of layer thickness with femtosecond time resolution.

Here, we use ultrafast optical microscopy (UOM)^12^,^13^ to measure ultrafast carrier dynamics in atomically thin single molybdenum disulfide flakes grown by chemical vapor deposition (CVD). By tuning the probe photon energy through the MoS_2_ band gap (both indirect and direct), our UOM measurements show that conduction and valence band states are rapidly populated on a sub-picosecond (ps) time scale in a MoS_2_ monolayer after photoexcitation at 3.1 eV, consistent with previous work^14^,^15^,^16^,^17^,^18^. Pump fluence-dependent measurements reveal that subsequent carrier relaxation in our samples is primarily due to surface-related defects and trap states, not the Auger processes observed in previous measurements on MoS_2_ and other semiconductor nanosystems^12^,^18^,^19^,^20^,^21^,^22^,^23^. We also observed an increase in the carrier relaxation time with an increase in the number of MoS_2_ layers, likely due to the well known layer-dependent changes in the electronic structure^16^,^24^. Our UOM measurements of photon energy-and-fluence-dependent carrier dynamics in single MoS_2_ flakes thus shed light on carrier relaxation in these nanosystems, which should influence future optoelectronic and nanoelectronic applications^15^,^16^,^25^.

## Experimental setup and sample description

**Figure 1:**
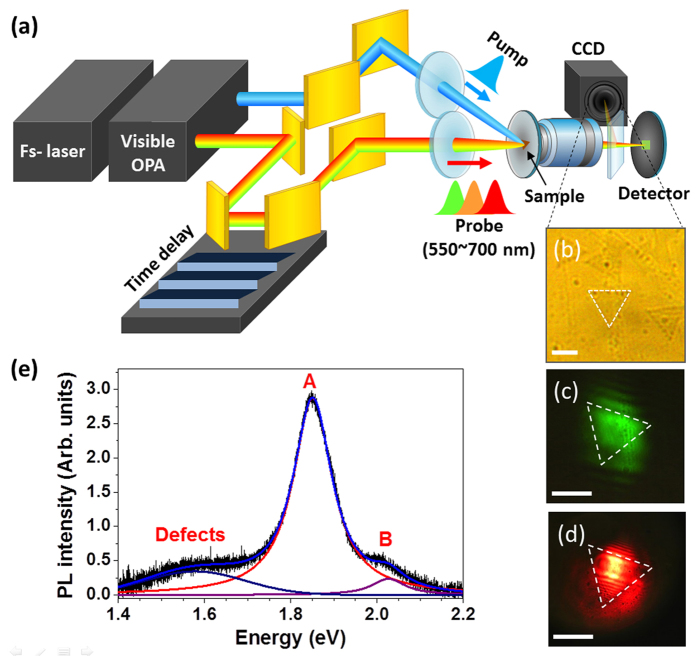
Experimental setup for ultrafast optical microscopy, as well as microscopic images and photoluminescence spectra of single MoS_2_ flakes. (**a**) Schematic of the ultrafast optical microscopy (UOM) system. (**b**) A microscope image of single MoS_2_ flakes taken with our UOM setup. The white dashed triangle outlines a single MoS_2_ flake. (**c,d**) Transmitted probe images at 550 nm and 650 nm, respectively, with the pump blocked. The scale bar is 10 μm. (**e**) Photoluminescence spectrum from a monolayer MoS_2_ flake. The A exciton peak (1.85 eV), B exciton peak (2.02 eV), and a peak due to defects (1.57 eV) are marked, and the solid lines show Gaussian fits to the data.

UOM is based on conventional optical pump-probe spectroscopy, with the incorporation of tightly focusing lenses or objectives to directly monitor samples with high spatial resolution (Fig. 1(a)). In our system, a 100 kHz regeneratively amplified laser producing 50 femtosecond (fs), 10 μJ pulses at 800 nm is used to pump a visible optical parametric amplifier (OPA)^21^. The signal wavelength from the OPA was tuned to generate linearly polarized probe pulses (with polarization perpendicular to the pump pulses) from 550 nm (2.24 eV) to 700 nm (1.77 eV), covering the bandgap (both indirect and direct) of MoS_2_, as well as the well-known A and B exciton transitions^16^,^26^. The MoS_2_ flakes were photoexcited at 400 nm (3.1 eV), well above the bandgap, with pump fluences (*F*) ranging from ~95–500 μJ/cm^2^. The pump and probe spot diameters were 63 μm and 30 μm, respectively, which fully covered a single MoS_2_ flake without overlapping other flakes. The normalized photoinduced change in transmission (ΔT/T), which is directly proportional to the photoexcited carrier population at a given point in time and space^15^,^21^,^27^, is measured at the detector. Real time monitoring of both focused spots and the sample position using a CCD camera with a 50X objective and accompanying beam expander allows us to selectively measure ultrafast carrier dynamics in individual nanomaterials, removing the detrimental influence of inhomogeneous broadening^12^,^13^.

Images of the atomically thin monolayer (0.6 nm in thickness) MoS_2_ flakes taken with our UOM system are shown in Fig. 1(b–d). Briefly, MoO_3_ particle-covered SiO_2_ substrates were placed in a furnace with a N_2_ gas flow, and heated up to 850 °C in the presence of a sulfur source. The as-grown monolayer MoS_2_ flakes were then transferred onto MgO substrates for optical measurements via the commonly used polymer supported method (PMMA). An image taken in our system shows the triangular shape of typical monolayer MoS_2_ flakes, in which each side is ~15–20 μm in length (Fig. 1(b)). Figure 1(c,d) show images of the transmitted probe beam at 550 nm and 650 nm (with the pump blocked), confirming that the probe beam completely covers single triangular MoS_2_ flakes. We also examined double monolayer and multiple layer MoS_2_ flakes in our experiments. Double monolayer MoS_2_ flakes consist of overlapping triangular monolayer flakes with different orientations and thus appear flower shaped (please see Supplementary Figure S1), and multiple layer flakes consist of overlapped monolayers and bilayers, possibly including trilayers, causing them to appear star or flower shaped. Double and multiple layer flakes have many corners and grain boundaries, allowing us to distinguish between flakes with different layer thicknesses in our UOM system. All experiments were performed at room temperature. Photoluminescence (PL) spectra were also taken on all samples. Figure 1(e) shows the PL spectrum for monolayer MoS_2_ at room temperature, revealing the A exciton peak at ~1.85 eV and the B exciton peak at ~2.02 eV. The small lower energy peak at 1.57 eV is due to defects^28^.

## Results

**Figure 2:**
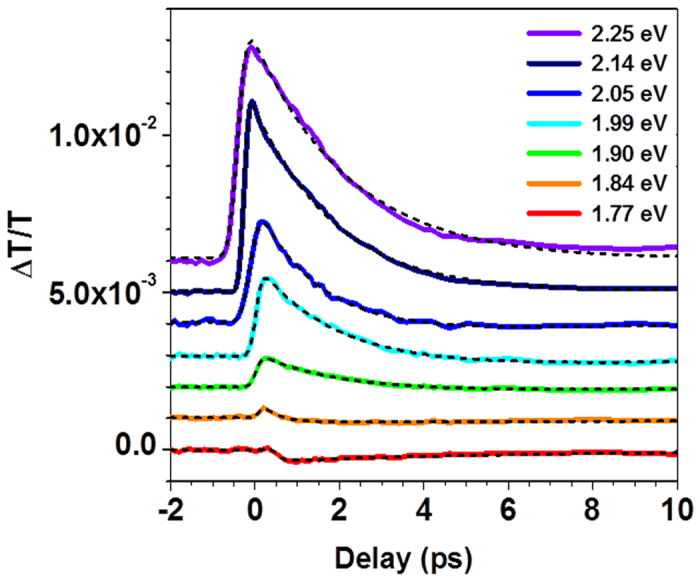
Ultrafast carrier dynamics in single monolayer MoS_2_ flakes for different probe photon energies. Photoinduced changes in transmission for various probe photon energies in a single flake of monolayer MoS_2_. Black dashed lines denote exponential curve fits for various probe photon energies. The curves are displaced vertically for clarity.

UOM experiments were performed on individual MoS_2_ flakes with different thicknesses as a function of the probe photon energy, with the pump fluence initially fixed at 320 μJ/cm^2^ (producing an initial photoexcited carrier density of *n*~10^14^ cm^−2^, calculated using the absorbance in ref. 29). The time-dependent ΔT/T signal obtained from a single flake of monolayer MoS_2_ for selected photon energies is depicted in Fig. 2. The photoinduced change in transmission reaches a maximum within ~500 fs, after which the subsequent fast relaxation occurs on a time scale of a few ps. Photoinduced changes in transmission above and near the bandgap (probe photon energy 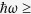 1.9–2.25 eV) on a single flake of monolayer MoS_2_ are initially positive, and the ΔT/T signal amplitude increases with the probe photon energy. This is expected since there is a greater population of carriers at higher energies than lower energies (i.e., the initial photoexcited carrier population at 3.1 eV loses carriers to non-radiative recombination as carriers relax to lower energies). This positive signal rapidly decays within a few picoseconds (ps), followed by a small negative signal at lower photon energies. When decreasing the probe photon energy to 1.84 eV, near the A excitonic peak (~1.85 eV), we observe a positive peak that decays within a few hundred fs, with a residual negative signal at longer time delays. At even lower photon energies (1.77 eV), the signal is always negative, recovering on a longer time scale of tens of picoseconds.

Physically, the pump pulse initially photoexcites carriers in the MoS_2_ monolayer to higher energy states (primarily the C exciton states near the Γ point^14^,^16^,^29^,^30^). The initial photoexcited carrier density is comparable to the Mott density of ~*n*~10^14^ cm^−2^, suggesting that the Coulomb interactions between carriers are largely screened and the photoexcited electrons and holes are no longer bound into excitonic states^18^,^31^. The non-equilibrium electrons rapidly scatter towards the conduction band minimum at the K point^14^,^15^,^16^, leading to a positive signal for probe photon energies above the bandgap (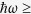 1.9 eV) through state filling (Fig. 2). In contrast, the photoexcited holes have a relatively low probability of scattering to the K point^14^, since the valence band maximum is comparable in energy at both the Γ and K points (see, e.g., Fig. 1 in ref. 30). Therefore, a significant fraction of the non-equilibrium holes likely remain near the Γ point at early times, leading to the negative peak observed within the first few ps at 1.77 eV (through induced absorption). Finally, the initial positive peak observed at 1.84 eV is likely due to the interplay between bandgap renormalization, which has recently been shown to influence the observed dynamics at these carrier densities^18^, and incompletely screened excitons.

At the highest probe photon energies examined here (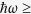 2.14 eV), the positive ΔT/T signal recovers within ~2 ps (obtained from exponential curve fits to the data) as the photoexcited electrons relax to lower energies. At lower photon energies (1.9 eV 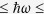 2.05 eV), we observe a small negative signal after the initial fast relaxation that recovers within 4–5 ps. This is likely due to induced absorption of holes near the Γ point; ref. 32 demonstrated that after photoexciting a MoS_2_ monolayer at 3.1 eV, photoluminescence at the A exciton peak appears and reaches a maximum within ~5 ps (limited by their experimental resolution). This indicates that photoexcited holes scatter from the Γ point to the K point within this timescale, agreeing well with our data. Finally, the negative signal observed on longer timescales for 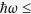 1.84 eV can be attributed to induced absorption from electrons that have relaxed into surface-related defect or trap states^16^,^23^,^33^, which should have an appreciable density based on the magnitude of the 1.57 eV peak in our PL data (Fig. 1(e)) (further supported by the fluence-dependent data discussed below). Our curve fits indicate that these carriers relax within ~30 ps.

**Figure 3:**
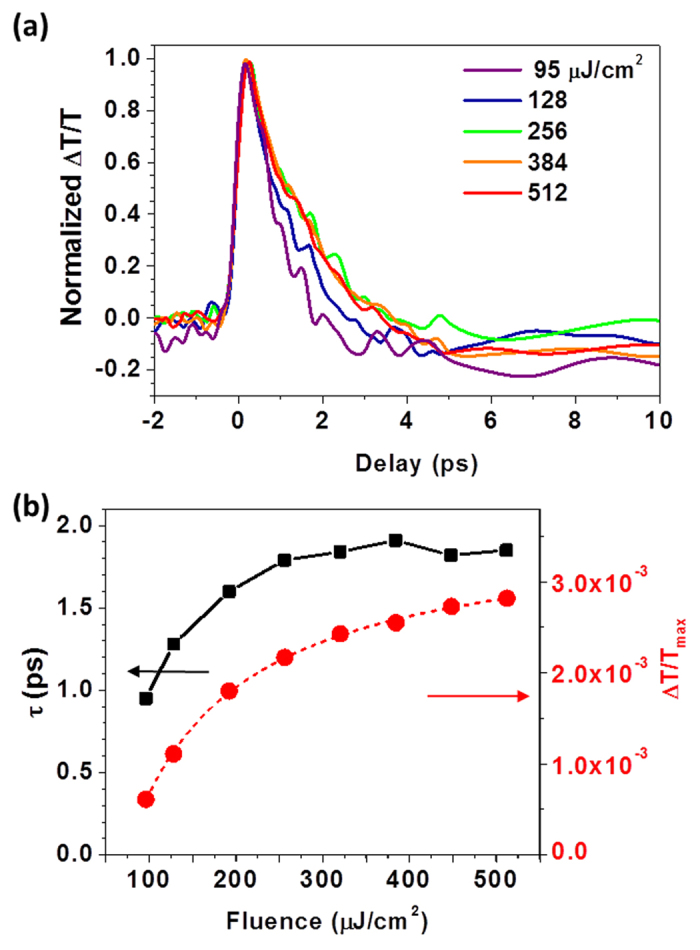
Pump fluence dependence of ultrafast carrier relaxation in MoS_2_ monolayers. (**a**) Pump fluence dependence of the photoinduced changes in transmission near the bandgap, 1.91 eV, for a MoS_2_ monolayer. (**b**) Plots of the decay time constant and maximum differential transmission signal extracted from (a) as a function of the pump fluence. The dashed red line represents the fit to the ΔT/T signal that allows us to extract *F*_*sat*_ = 51.5 μJ/cm^2^ 40,41.

We also examined the dependence of the observed carrier dynamics on pump fluence for the single MoS_2_ monolayer flake, in a regime (*F*~95–500 μJ/cm^2^) that has not been extensively studied^17^,^18^,^34^,^35^. Figure 3(a) shows the normalized transmission changes for various pump fluences at a probe photon energy of 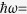 1.91 eV, revealing that the initial decay time τ increases as a function of pump fluence. This is quantified in Fig. 3(b), which shows that τ increases by a factor of two and the maximum value of ΔT/T increases by more than a factor of three over the measured fluence range, saturating at the highest fluences (likely due to state filling near the band edge). This dependence of τ on *F* is the opposite of what one would expect for interband Auger recombination, a three-carrier non-radiative process that often dominates carrier relaxation at high fluence in semiconductor nanostructures^19^,^20^,^21^,^22^,^23^,^27^. This is also inconsistent with Auger-induced capture into defect states, as described in refs.^19^,^27^, which predicts the carrier lifetime to decrease with increasing fluence.

Instead, the observed fluence dependence is likely due to one or both of two processes. Ref. 17 observed a similar increase in the carrier lifetime with fluence in few-layer MoS_2_ over a comparable fluence range. This was attributed to the hot phonon effect, in which a large phonon population, created by the relatively high temperature and pump fluence, inhibits carrier relaxation to lower energies (which typically occurs through phonon emission^21^), since the transient phonon temperature becomes comparable to that of the non-equilibrium electron distribution^36^,^37^. Carrier relaxation into surface-related defect and trap states can also influence the observed fluence dependence. These defect states are known to affect the electronic and optical properties of transition metal dichalcogenide monolayers^28^,^38^, and the relatively large defect-related PL peak in Fig. 1(e) suggests that they will also influence the properties of our MoS_2_ monolayers. For low pump fluences (*F* < 250 J/cm^2^), photoexcited carriers can quickly relax into these defect states (within τ ~ 1–1.5 ps). However, the defect states will be filled at high pump fluences, causing ΔT/T to saturate and τ to increase (since photoexcited carriers in states near the band edge that are examined by the probe photons have nowhere else to relax to before recombining on longer timescales) (Fig. 3(b))^16^,^39^. The data in Fig. 3 shows that τ does not change significantly after *F*~256 μJ/cm^2^ (corresponding to *n*~8 × 10^13^ cm^−2^), suggesting that the defect states are filled for these higher pump fluences. We can gain more insight by fitting our fluence-dependent ΔT/T data (Fig. 3(b)) to obtain the saturation fluence *F*_*sat*_^40^,^41^. This gives *F*_*sat*_ = 51.5 μJ/cm^2^, corresponding to a saturated carrier density of *n*_*sat*_~1.6 × 10^13^ cm^−2^. Ref. 38 indicates that the defect density in CVD-grown MoS_2_ monolayers is ~10^13^ cm^−2^, very close to *n*_*sat*_. This supports the idea that defect and trap states dominate carrier relaxation in our MoS_2_ monolayers, although we cannot rule out contributions from the hot phonon effect. Temperature-dependent measurements^17^ and tuning the probe wavelength to the defect state PL peak at 1.57 eV could enable us to gain more insight into this in the future. It is worth noting that we observed similar trends at other probe photon energies (e.g., 1.99 eV and 2.25 eV).

**Figure 4:**
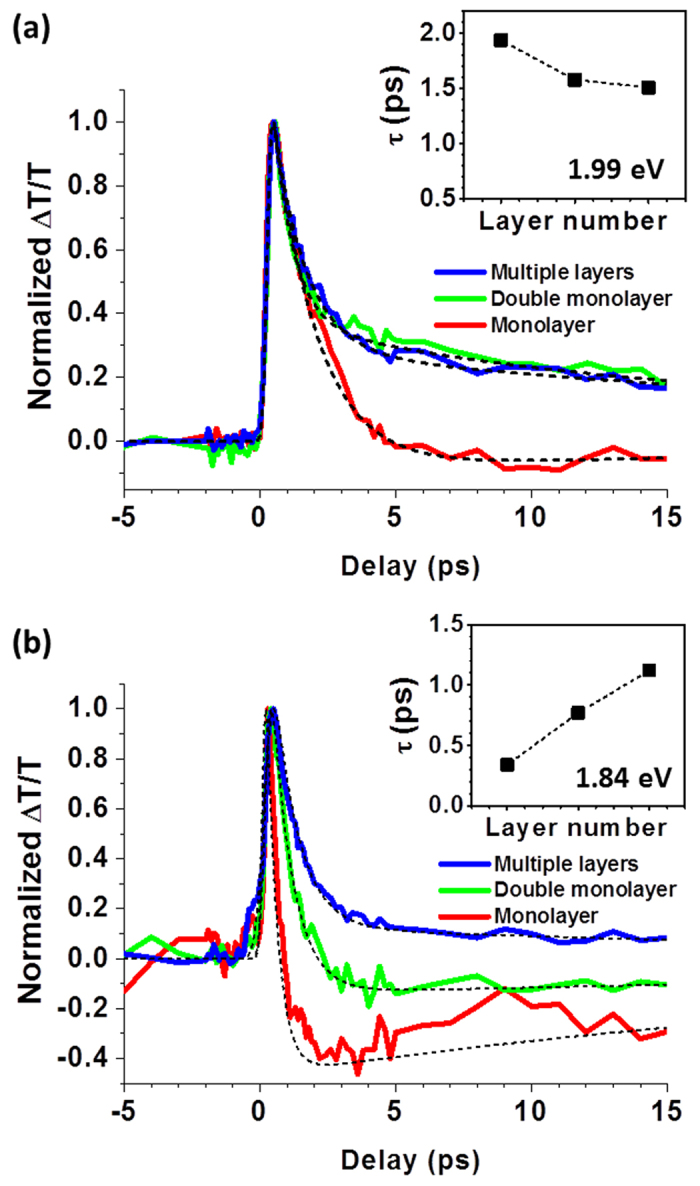
Ultrafast carrier dynamics in MoS_2_ flakes at different probe photon energies for different layer thicknesses. (**a**) Normalized photoinduced changes in transmission at 1.99 eV for different MoS_2_ samples. The monolayer sample is identical to that described in Figs 1 and 2. The ‘double monolayer’ sample consists of monolayers in contact with one another, while the multilayer sample consists of multiple layers of MoS_2_ grown on top of one another. (**b**) Normalized photoinduced changes in transmission at 1.84 eV for the different MoS_2_ samples. The insets show the dependence of the decay time constant on the layer thickness.

Finally, the well-known variation of the electronic structure in MoS_2_ with number of layers (e.g., a shift from an indirect gap in multilayer MoS_2_ to a direct band gap in monolayer MoS_2_) makes it worthwhile to investigate carrier relaxation in samples with different layer configurations. Photoinduced changes in transmission were measured in isolated MoS_2_ monolayers, double monolayers, and multilayer MoS_2_ using various probe photon energies. Figure 4(a) depicts the ΔT/T signals using a probe photon energy of 1.99 eV. The signal increases with the number of layers because there are more atoms within the excitation spot to absorb the incoming pump photons. The time constant τ decreases with layer thickness (Fig. 4(a) inset), consistent with previous measurements^16^. At 1.84 eV, however, the initial ΔT/T signal remains positive for the multilayer MoS_2_ sample, even at long time delays (>10 ps) (Fig. 4(b)), in contrast with the rapid transition to a negative value for the isolated monolayer, as discussed earlier. In addition, τ increases with the number of MoS_2_ layers (Fig. 4(b), inset). These trends are due to the transition from a direct to an indirect bandgap with increasing layer thickness^4^,^5^. As described above, in the MoS_2_ monolayer, the 1.84 eV probe photons initially probe carrier dynamics near the conduction band edge at the K point, as described above. In contrast, in the multilayer sample the 1.84 eV probe photons examine a phonon-assisted absorption from the valence band maximum at the K point to the conduction band minimum between the Γ and K points. The separation of the electron and hole populations in momentum space, as well as the reduced influence of the surface as the number of layers increases, both increase τ for the multilayer sample. Our UOM results thus can be linked to the changes in the MoS_2_ electronic structure with the number of layers.

## Conclusion

In conclusion, we have used ultrafast optical microscopy to measure carrier dynamics in single MoS_2_ flakes with femtosecond temporal resolution and micron spatial resolution. By tuning the probe photon energy through the bandgap of the MoS_2_ flakes, we can obtain temporally and spectrally resolved measurements of carrier dynamics that establish the timescale for carrier relaxation after pump photoexcitation. Furthermore, we observed a variation in the carrier dynamics with pump fluence, primarily due to relaxation into surface-related defect and trap states that eventually saturate at high fluences. Finally, our measurements on samples with different numbers of MoS_2_ layers are consistent with the known changes in the band structure with layer thickness. Overall, our UOM studies on monolayer and few-layer MoS_2_ provide important insight into the physics of these interesting 2D systems.

## Additional Information

**How to cite this article**: Seo, M. *et al.* Ultrafast optical microscopy of single monolayer molybdenum disulfide flakes. *Sci. Rep.* **6**, 21601; doi: 10.1038/srep21601 (2016).

## Supplementary Material

Supplementary Information
