# Supplementary material for: Ultrafast Optical Microscopy of Single Monolayer Molybdenum Disulfide Flakes
Source: Sci Rep. 2016 Feb 15;6:21601. doi: 10.1038/srep21601 (PMC4753495; doi:10.1038/srep21601)
Supplement: Supplementary Information [file srep21601-s1.pdf]

Supplementary Information for Ultrafast Optical Microscopy of Single Monolayer Molybdenum Disulfide Flakes

Minah Seo[1,2], Hisato Yamaguchi[3], Aditya D. Mohite[3], Stephane Boubanga-Tombet[1], Jean-Christophe Blancon[4], Sina Najmaei[5], Pulickel M. Ajayan[5], Jun Lou[5], Antoinette J. Taylor[1], and Rohit P. Prasankumar[1,*]

[1]*Center for Integrated Nanotechnologies, Los Alamos National Laboratory, Los Alamos, NM, 87545, USA*

[2]*Sensor System Research Center, Korea Institute of Science and Technology, Seoul, Republic of Korea*

[3]*Materials Synthesis and Integrated Devices, Materials Physics and Applications Division, Los Alamos National Laboratory, Los Alamos, NM 87545, USA*

[4]*Physical Chemistry and Applied Spectroscopy, Chemistry Division, Los Alamos National Laboratory, Los Alamos, NM 87545, USA*

[5]*Department of Materials Science and NanoEngineering, Rice University, Houston, Texas 77005, USA*

*rpprasan@lanl.gov

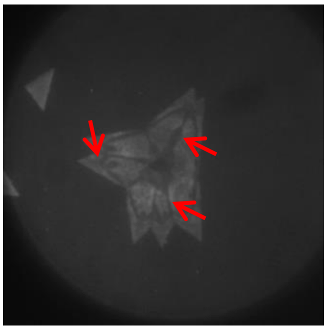

Figure S1. Fluorescence microscope images of a flower-shaped double layer of MoS_2, showing gaps in the fluorescence at the boundaries between grains (indicated by the red arrows). More detail on the properties of similar samples is given in reference S1.

S1. S. Najmaei et al, "Vapour phase growth and grain boundary structure of molybdenum disulphide atomic layers," *Nature Materials* **12**, 754 (2013).
